# Stroke and cardiovascular risk factors among working-aged Finnish migraineurs

**DOI:** 10.1186/s12889-021-11006-1

**Published:** 2021-06-07

**Authors:** Marja-Liisa Sumelahti, Merika S. Sumanen, Kari J. Mattila, Lauri Sillanmäki, Markku Sumanen

**Affiliations:** 1 Faculty of Medicine and Health Technology, Tampere University, 33014 Tampere, Finland; 2 Department of Public Health, Turku University Hospital, Turku, Finland

**Keywords:** Migraine, Women, Ischemic stroke, Myocardial infarction, Triptan use

## Abstract

**Background:**

The aim of our study was to evaluate the risk for comorbid cardio- and cerebrovascular diseases in the working-aged migraine population of Finland.

**Methods:**

A total of 1505 cases who reported diagnosed migraine and 3010 controls from a cohort of 11,596 cases in the Finnish Health and Social Support Study were included. The study material was linked with two registers. ICD diagnoses I63 for ischemic stroke (IS), I21 − I22 for acute myocardial infarction (AMI), and G43 for transient ischemic attack (TIA) among study participants were drawn from the national Finnish Care Register for Health Care at the follow-up in 2012. Reimbursed triptan prescriptions were drawn from the national Social Security Institution (SII) data. The self-reported vascular risk factors were hypertension, high cholesterol values, any diabetes, myocardial infarction, stroke, and TIA. Odds Ratios (OR) with 95% confidence (95% CI) intervals were assessed for diagnosed stroke, myocardial infarction, and TIA.

**Results:**

Migraineurs were mostly female (82%) and ≥ 54 years old (62%). Triptans were reimbursed among 34.7% of migraineurs. A self-reported hypertension (21%), high serum cholesterol (38%), and any diabetes (7%) were more common among migraineurs vs controls (*p* < 0.05). There was no risk for AMI. The risk for TIA (OR 3.20, 95% CI 1.45–7.05) and IS (2.57, 95% CI 1.28–5.17) among migraineurs vs controls remained high after adjustment for self-reported hypertension, obesity, and smoking. The risk was higher among women in two groups ≥54 years (3.25, 95% CI 1.35–7.84 and 5.0, 95% CI 1.94–12.89, respectively). The average age for IS in migraine was 57.5 years and for TIA 58.2 years among women, and 52.8 years and 50.3 years among men, respectively.

**Conclusion:**

Cardiovascular risk should be screened in the aging migraine population, and hormonal and other migraine-related risk factors should be considered, especially among women. Efficacious attack treatment with triptans should be offered to migraine patients who do not show contraindications.

## Background

Migraine affects up to 15% of adults and peaks among women and the working-age population [1]. A combination of attacks with aura (MA) or without aura (MO) at varying frequency may occur, and the changing vascular risk factors during the lifespan challenge the assessment of the migraine-related risk for comorbidities, such as vascular diseases, in long-term studies [2].

Population-based and prospective studies have consistently reported an increased risk of ischemic stroke and cardiovascular disease in patients with overall migraine [3]. The exact mechanisms by which migraine might increase the risk are not known. Understanding of cardio- and cerebrovascular risk in migraine has become more precise over the last decades, pointing at MA, female gender, and other independent risk factors [4–6]. The connection between migraine, stroke, and other cardiovascular diseases relates also to higher cardiovascular risk factors in migraineurs than in people without migraine [7]. Furthermore, genetic overlap exists between migraine, ischemic stroke [7], and coronary artery diseases [8], pointing to an overlap with large-artery and cardioembolic subtypes and MO [7].

Stroke is considered a rare event among patients with migraine [6]. In a subgroup of women under 45 years old, there is an increased risk for stroke that is exacerbated by oral contraceptive use, smoking, and high blood pressure [4, 6]. Less information is available about risk of stroke after the age of 50 years and among elderly migraineurs.

The aim of our study was to evaluate the risk for comorbid cardio- and cerebrovascular diseases and related self-reported risk factors in the working aged migraineur population in Finland. Cases and controls are drawn from the Finnish Health and Social Support Study. Diagnostic data are based on ICD-coded diagnoses drawn from the national Finnish Care Register for Health Care.

## Methods

The participants and information were drawn from the Health and Social Support (HeSSup) Study in 1998, 2003, and 2012 [9]. HeSSup was a prospective follow-up study that ran from 1998 to 2012 on the psychosocial health of the Finnish working-age population, and it included 33 items regarding health and several common disorders.

**Fig. 1:**
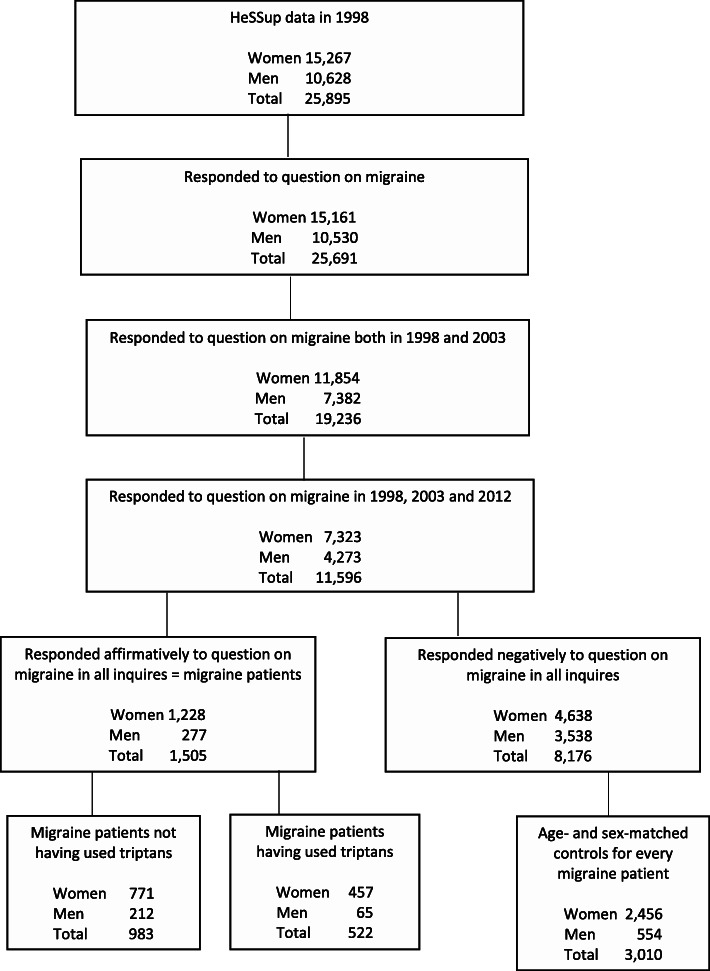
Flow chart of the study population

The first questionnaire in 1998 was sent to a random sample of 64,797 working-age individuals drawn from the Finnish Population Register in four five-year age groups (20–24, 30–34, 40–44 and 50–54) [9]. The response rate was 40%. A total of 25,691 (99.2%) responded to the question on migraine in 1998, and up 11,596 had responded to the question on migraine in all three questionnaires. According to the non-response analysis in 1998, respondents and non-respondents were comparable with respect to the most important demographic variables, including gender and age distribution. Moreover, the differences in physical health between participants and the general population were minor [9, 10]. Definitions of the study groups in the three surveys are shown in Fig. 1.

Finnish residents are offered universal healthcare, and the quality of service in Finnish publicly funded healthcare is considered good [11]. The statutory National Health Insurance (NHI) scheme covers all Finnish residents, and it is run by the Social Insurance Institution (SII). The International Headache Society’s International Classification of Headache Disorders (ICHD) criteria for migraine (https://www.ichd-3.org) are followed by doctors in Finland. Migraine treatment is guided by national Current Care Guidelines developed and updated by the Finnish Medical Society [12].

The data were linked with the Finnish Care Register for Health Care (Health Care Register) by using the participants’ personal identity code given to all Finnish citizens as a key (https://vrk.fi/en/personal-identity-code1). ICD-10 codes I63 for Ischemic Stroke (IS), G45 for Transient Ischemic Attack (TIA), and I21 and I22 for Acute Myocardial Infarction (AMI) were searched for. Linkage to registers of the Finnish Social Insurance Institution by personal identity code was used to study the prescribed and reimbursed triptans.

The inclusion criterion for being a migraine patient was self-reported migraine, based on whether a doctor had told the respondent that he/she has or has had a migraine in all three HeSSup questionnaires [9]. The control group from the same data pool consisted of two age- and sex-matched controls for every migraine patient among those who had responded negatively to the questions on migraine. Approximately 1% of those having responded negatively to the questions on migraine were excluded from the control group if they had been prescribed triptans. The data including cases and controls were next incorporated for self-reported cardio- and cerebrovascular risk factors for hypertension, high serum cholesterol, any diabetes, myocardial infarction, angina pectoris, ischemic stroke, or other cerebrovascular disorders, based on whether a doctor had told the respondent that he/she has or has had such a disorder. Flow chart of the study population is shown in Fig. 1.

In the present HeSSup migraine data, the occurrence of self-reported disorders under study was assessed in the migraine and control groups by using the Chi-squared test to study statistical differences. Univariate conditional logistic regression analysis was performed in the two oldest age groups in 2012 (54–58 and 64–68), based on reported cardiovascular endpoints occurring most commonly among older people [13]. Odds Ratios (OR) with 95% confidence intervals (CI) were assessed for diagnoses drawn from the National Institute for Health and Welfare for AMI (I21 − I22), for ischemic stroke (I63), and for TIA (G45) in the migraine and control groups. This analysis was applied to study crude OR and also OR adjusted (aOR) for self-reported hypertension, obesity (BMI ≥ 30), and smoking. Finally, the average age at the cardiovascular endpoints were assessed among migraineurs and controls, group difference was assessed by using T-test and *p*-value ≤0.05 was considered significant.

The data were analyzed using IBM SPSS Statistics 23 (IBM Corporation, Armonk, New York, USA) and SAS 9.4 (SAS Institute Inc., Cary, NC, USA).

## Results

We included 1505 migraineurs (female to male ratio 1228/277) and 3010 controls among the 11,596 responders in 2012. The occurrence of migraine was 13%, and most of all migraine patients (62%) belonged to the two oldest (54–58 and 64–68 years) age groups in 2012. Triptans were reimbursed by the SII in 522 cases (34.7%) among all migraineurs and equally in age groups up to 58 years, but less often (16%) in the oldest age group of 64–68 years (Table 1).

**Table 1:** Age and gender distribution among migraine patients (*n* = 1505) by triptan use and among controls (*n* = 3010) in 2012

	Migraine patients				Controls	
	Not used triptans		Used triptans			
	n	%	n	%	n	%
Gender						
Women	771	78.7	457	87.5	2456	81.6
Men	212	21.6	65	12.5	554	18.4
Born on years						
1944–1948 (age 64–68)	403	41.0	85	16.3	976	32.4
1954–1958 (age 54–58)	279	28.4	161	30.8	880	29.2
1964–1968 (age 44–48)	164	16.7	137	26.2	602	20.0
1974–1978 (age 34–38)	137	13.9	139	26.6	552	18.3

The self-reported occurrence of hypertension in migraine was 21.3% (all cases) vs 15.5% in controls, the difference was statistically significant. Statistical differences between the groups were found also for high serum cholesterol (38.2% vs 33.0%), stroke (1.4% vs 0.5%), and other cerebrovascular disorders (4.5% vs 1.7%). No difference was found for any diabetes, myocardial infarction, or angina pectoris (Table 2).

**Table 2:** Self-reported vascular comorbidies among migraine patients by triptan use and among controls in 2012

	Migraine patients						Controls		
	Not used triptans		Used triptans		All				
	n	%	n	%	n	%	n	%	p-value
Hypertension	237	24.3	83	16.0	320	21.3	462	15.5	< 0.001
High cholesterol value	415	42.4	160	30.9	575	38.2	991	33.0	< 0.001
Diabetes	81	8.3	25	4.8	106	7.0	173	5.8	0.073
Myocardial infarction	19	2.0	3	0.6	22	1.5	35	1.2	0.333
Angina pectoris	37	3.8	4	0.8	41	2.7	57	1.9	0.057
Stroke	14	1.4	7	1.3	21	1.4	14	0.5	< 0.001
disorder	51	5.2	16	3.1	67	4.5	51	1.7	< 0.001

In the univariate conditional logistic regression analysis in the age groups 54–58 and 64–68 years, ischemic stroke (OR 95% CI: 2.57, 1.28–5.17) and TIA (OR 95% CI: 3.20, 1.45–7.05) were statistically significant among migraine patients. This is, however, valid only among women (OR 95% CI: 5.00, 1.94–12.89, and 3.25, 1.35–7.84, respectively). No risk was seen for AMI. Adjusting for self-reported hypertension, obesity and smoking did not have any notable influence (Table 3).

**Table 3:** Conditional logistic regression analysis (ORs with 95% CI) in migraine for Health Care Register diagnoses I21–22, I63 and G45 in age groups 54–58 and 64–68 years (*n* = 928)

	Crude	Adjusted ^a^
Endpoint (ICD-10 code)	OR (95% CI)	aOR (95% CI)
Acute Myocardial Infarction (I21–22)		
All migraineurs	1.63 (0.75–3.55)	1.37 (0.62–3.01)
Women	1.85 (0.69–4.99)	1.46 (0.53–3.98)
Men	1.33 (0.38–4.73)	1.22 (0.34–4.35)
Ischemic Stroke (I63)		
All migraineurs	2.57 (1.28–5.17)	2.24 (1.10–4.56)
Women	5.00 (1.94–12.89)	4.32 (1.65–11.29)
Men	0.75 (0.20–2.83)	0.69 (0.18–2.60)
TIA (G45)		
All migraineurs	3.20 (1.45–7.05)	3.32 (1.46–7.58)
Women	3.25 (1.35–7.84)	3.35 (1.32–8.50)
Men	3.00 (0.50–17.95)	3.09 (0.52–18.53)

^a^adjusted for hypertension, obesity and smoking

The average age at diagnosed TIA was lower among migraineurs compared to the controls (56.4 vs 58.7 years, *p* = 0.004). In ischemic stroke, the corresponding ages were similar, 56.5 vs 57.1 years (*p* = 0.053) and in myocardial infarction, 55.3 vs 57.7 years (*p* = 0.849).

## Discussion

Epidemiological and genetic overlap exists between migraine and arteriovascular diseases. Age-specific stroke risk in migraine points at young women with the aura subtype, while less is known for the risk in the aging population. In our study, an increased risk for ischemic stroke was observed among women and in older age-groups. Among men, no increased risk was found. This finding can be attributed to the low numbers, as men are less likely to be migraineurs compared to women. In line with other reports, our data show a 13% migraine prevalence in the working-age population [1]. Here, the occurrence for cerebrovascular disorders, hypertension, and high cholesterol associated with migraine more often than among the controls.

In our total migraine cohort, the overall two-fold risk for ischemic stroke corroborates other reports [6, 7]. The occurrence of ischemic stroke in migraine is low, and the concept of stroke contains two separate disorders [14]. The incidence of a true migrainous infarction is very low, accounting for less than 1% of all ischemic strokes [15]. Based on the data from the Health Care Register, we were not able to separate cerebral infarctions occurring during the course of a typical migraine with an aura attack (migrainous infarction) from cerebral infarctions of another cause coexisting with migraine (migraine-related stroke). The reliability of the stroke diagnosis in migraine is dependent on the accurate diagnosis of migraine, which may be suspected to be underreported along with under-recognized migraine [16, 17]. In our data, the increased five-fold risk for ischemic stroke and more than three-fold risk for TIA among women in age-groups over 54 years are significantly higher than the risk observed among men. The risk among women remained high after adjusting for smoking, obesity, and hypertension, which is regarded as the most important risk factor for stroke [18]. A generally lower stroke risk among men has been shown in several studies [19], but there are few reports on the gender-specific stroke risk or the risk in older age-groups in migraine [20, 21]. Among the known stroke risk factors and health behaviors in migraine are smoking, obesity, use of hormonal replacement therapy, and the use of the combined oral contraceptive [22, 23]. These factors have been reported mainly among younger women, and the risk has been generally related to migraine with aura [6, 7]. However, these risk factors may be present in both younger and older female age-groups, and a combination with other vascular risks may contribute to the risk [2]. The frequency of migraine has also been shown to be a risk factor for stroke among women aged 25–42 years, as shown in the Women’s Health Study [24], but this has not been identified in older age-groups. Stroke risk factors and migraine subtypes and patterns remain important confounders in future studies concerning the overall risk and especially the age-specific gender risk.

In line with other epidemiological studies, the reported migraines in our data were associated with comorbid hypertension, high cholesterol, and angina pectoris. Results from the genome-wide association data suggest that shared biological processes contribute to the risk of migraine and coronary artery disease, and they point to the genes controlling for endothelial dysfunction and insulin homeostasis [5]. However, no risk for AMI in the Health Care Register data was observed, corroborating results from a meta-analysis [6]. Also, our results on low female coronary artery disease risk are in contrast with the results among the participants of the Women’s Health Study, where an association with migraine and any cardiovascular disease, coronary events, and cardiovascular mortality was shown [24]. The vascular disease burden is increasing globally, and in spite of these discrepancies, there is a need to increase awareness in the recognition, prevention, and treatment of common vascular risk factors in migraine and coronary artery disease. Evidence that insulin resistance may be linked to endothelial dysfunction and vascular disease risk [7, 8] points to the need for lifestyle improvements among at-risk individuals. Although hypertension is regarded as the most important risk factor for stroke,21 we observed no increase in risk after adjustment for reported hypertension. The occurrence of reported hypertension was significantly higher in migraineurs (21%) compared to the controls (15%), and this corroborates other studies [25, 26]. We also observed higher cholesterol values among migraineurs (38% vs 33% in controls). We did not control for antihypertensive drug use, which may affect the stroke risk [27], nor for the use of cholesterol-lowering drugs.

Migraine was self-reported, based on whether a doctor had told the patient of such a diagnosis. The self-reporting of migraine is used in several studies and is considered reliable [28]. The classification of a given primary headache disorder subtype may change, however, and the diagnostic certainty of other conditions may vary according to the doctor who made the diagnosis [29]. Moreover, it is possible that migraine is underdiagnosed in the present study. Tension neck headache is very common, but some of those individuals might be diagnosed as migraineurs if they were seen by specialists. The included cases had responded affirmatively to the inquiry on having migraine in all three questionnaires, and moreover, the proportion of cases with prescribed triptans may be considered appropriate [27]. When contraindications are followed, triptan treatment is not associated with the increased risk of stroke, even in the setting of overuse [30]. An unmet need in migraine treatment was observed here, as triptans were prescribed in less than half of all cases (34.7%). Nevertheless, the rate is higher than reported in American (18.3%) and Swedish studies, and it likely relates to the medication reimbursement practice in Finland [31, 32]. Triptans were used in all age groups, but less so in the oldest group and in cases of a reported cardiovascular condition.

We did not use diagnostic questions to verify the self-reported vascular disorders. The reported strokes and myocardial infarctions here may be regarded as reliable based on compatible observations and results after linkage to the Health Care Register ICD-10 diagnoses. Ischemic stroke diagnosis in hospital settings may be considered reliable due to the established diagnostic procedures, including brain tomography (CT) and MRI. There is a general risk for a missed stroke diagnosis more often in the case of posterior circulation stroke, including symptoms typical also in migraine attack, such as nausea, vomiting, and dizziness [16]. The risk for myocardial infarction was not increased among migraineurs in contrast to another study [33]. Misdiagnosis of AMI is unlikely, as it is explicitly diagnosed by serum troponin level and ECG change in Finnish hospitals. There are concerns in the case of TIA, as the inter-observer agreement in diagnosis has been low even in a neurological setting, and the specificity of the diagnosis is modest to low, reflected in a poor separation of TIA and mimics, particularly migraine with aura with its varied symptomatology [34]. This misdiagnosis is possible also in our data in cases where hospital-treated patients show both neurological symptoms and headache, or in cases of typical aura without headache. The ICHD-3 has recently refined the diagnostic criteria for aura to include positive symptomatology, which better differentiates aura from TIA. Recently proposed explicit diagnostic criteria for TIA may further improve the differential diagnosis [34].

A major strength in our data is the high response rates among the large group of migraineurs in the follow-up questionnaires. The possible differences in comorbidities other than cardiovascular comorbidities are not expected to influence on the results and conclusions of our study. Furthermore, exclusion of a suspected 1% false-negative migraine population according to triptan prescription data is not expected to interfere in this study. The limitations of the data are the low response rate in 1998 and the over-representation of older age groups. However, the careful non-response analysis indicated that respondents and non-respondents were comparable with respect to important demographic variables. In addition, the differences in physical health were minor. It is also possible that migraine patients have controlled their blood pressure and cholesterol level more often compared to other people, but this hardly had an influence on the results.

## Conclusions

The data in this study show that any migraine in the adult population associates with a high occurrence of known vascular risk factors. The ORs for diagnosed ischemic stroke and TIA are high in migraine. This paper is among the few studies pointing out higher ischemic stroke and TIA risk among women with migraine in older age groups. There is a need to anticipate vascular risk and recognize both sex-specific and general risk factors in all migraine age groups. Efficacious attack treatment with triptans should be offered more often to migraine patients who do not show contraindications.

## Data Availability

The data included in the manuscript are not publicly available per ethical guidelines of the protocol, but they are available via the corresponding author provided the appropriate ethical approvals are obtained.

## Abbreviations

MO: Migraine without aura
MA: Migraine with aura
IS: Ischemic stroke
TIA: Transient ischemic attack
AMI: Acute myocardial infarction
SII: Social Insurance Institution
HeSSup: Health and Social Support Study
NHI: National Health Insurance
ECG: Electrocardiogram
